# The prevalence of burnout and coping strategies among Palestinian health professionals: a cross sectional study

**DOI:** 10.3389/fpubh.2024.1477812

**Published:** 2024-12-18

**Authors:** Muna Ahmead, Nuha El Sharif, Abdallah Alwawi, Asma Hemeid, Motasem Ziqan

**Affiliations:** ^1^Faculty of Public Health, Al Quds University, Jerusalem, Palestine; ^2^Faculty of Health Professions, Al Quds University, Jerusalem, Palestine; ^3^Faculty of Medicine, Al Quds University, Jerusalem, Palestine

**Keywords:** professional burnout, coping strategies, nurses, health professionals, Palestine, physicians

## Abstract

**Introduction:**

During times of conflict, healthcare personnel face a heightened vulnerability to experiencing psychological problems such as burnout. The impact of conflict or wars on mental health professionals in Palestine and their strategies for managing these problems are currently not recognized. This study sought to assess the prevalence of burnout symptoms and coping strategies among healthcare workers in Palestine, in the context of the ongoing conflict and political violence.

**Methods:**

The study utilized a cross-sectional research design. Self-reported questionnaires, including the shortened version (9 items) of The Maslach Burnout Questionnaire and Brief COPE scales, were used to gather data. The relationship between the research variables and burn symptoms was investigated using Chi-squared test, Student’s *t*-test, Mann–Whitney, and Analysis of variance and multi-regression.

**Results:**

A total of 713 participants were recruited. The prevalence rates were as follows: burnout—(72.9%), emotional exhaustion (44.2%), depersonalization (9.8%), and low personal accomplishment (72.2%). The multivariate analysis found that those who worked more than 16 h per day and those who had 1–15 years of job experience were more likely to had emotional exhaustion. Active coping, substance use, informational support, and emotional support all reduced the likelihood of burnout while behavioral disengagement and self-blame increased the possibility of experiencing burnout. Finally, substance use decreased emotional exhaustion, increased depersonalization and enhanced personal accomplishment.

**Conclusion:**

The findings revealed a high prevalence of burnout among health professionals during wartime and political violence. As a result, health professionals are vulnerable to mental problems during political violence and they need immediate assistance in enhancing their mental wellbeing through psychological support, and comprehensive training in stress management.

## Introduction

War is a condition of armed enmity and violence between or within countries (1). The occurrence causes widespread destruction, massive human fatalities, and population displacement (2). Also, war has a significant impact on health, damaging the physical and mental well-being of persons living in war zones (3). Health services are frequently disrupted, resulting in a scarcity of medical supplies and healthcare personnel. Healthcare personnel, including physicians, nurses, pharmacists, and anesthetists, confront substantial challenges such as working in dangerous conditions, managing a large number of patients, and dealing with personal bereavement and emotional distress (4). Additionally, the destruction of healthcare infrastructure, such as hospitals and clinics, impairs the ability to provide adequate medical care (5). Furthermore, healthcare professionals frequently experience significant levels of stress and burnout, which are exacerbated by the ongoing threat to their safety and the excessive demand for medical services (6, 7).

Burnout is particularly prevalent in demanding professions like healthcare (8). Burnout is characterized by three distinct aspects: emotional exhaustion (EE), depersonalization (DP), and a decreased sense of personal accomplishment (PA) (9). It can arise from factors such as an overwhelming workload, a lack of control, inadequate incentives, a sense of isolation, a lack of justice, or conflicting values (10). Wars exacerbate these characteristics by causing extreme working conditions, prolonged stress, and traumatic events, all of which lead to occupational burnout among healthcare professionals (7, 11). Research has found that burnout rates among healthcare staff have reached critical levels, especially in areas experiencing ongoing violence (12, 13). Healthcare staff in Syria, Ukraine, and Libya experience significant emotional exhaustion and depersonalization due to the high number of victims and constant threats to their lives (12, 14). For example, a cross-sectional study done during the war in Ukraine indicated that emotional exhaustion (EE) and depersonalization (DP) were present in 31.6 and 33.4% of physicians, respectively, and in 17.9 and 18.2% of nurses (15).

The strategies employed by healthcare workers to manage stress might influence their vulnerability to burnout (16). Coping strategies are typically classified as problem-focused, emotion-focused, and avoidance-focused techniques (17). Problem-focused coping entails addressing the issue directly to minimize stress. Emotion-focused coping is the process of controlling emotions that develop as a result of a stressful circumstance (18). Avoidance-focused coping involves avoiding or distancing oneself from the situation (19). Research shows that problem-focused and emotion-focused coping are more successful in reducing burnout than avoidance-focused coping. For example, healthcare personnel who engage in regular physical exercise and seek social support had reduced rates of burnout (20).

In Palestine, the Israeli military has occupied the West Bank and Gaza Strip since 1967. Israel unilaterally withdrew all of its armed forces and settlers from the Gaza Strip in 2005. The Palestinian Ministry of Health (MOH), UNRWA, NGOs, and the private sector are all involved in the administration of healthcare services in Palestine, which also include primary, secondary, and tertiary levels (21). There are a total of 743 primary healthcare centers in Palestine, with 583 located in the West Bank and 160 in Gaza. The Palestinian National Institute of Public Health manages 81 hospitals, with 51 in the West Bank (including East Jerusalem) and 30 in Gaza (22). The continuous political violence in Palestine, especially after October 7, 2023, has had a significant impact on the health status in Palestine (23). The healthcare system encounters several obstacles, such as scarcity of medical resources, impaired infrastructure, and restricted availability of healthcare services (23). Healthcare professionals work under persistent danger, intensifying their stress levels and increasing the likelihood of experiencing burnout (24). For example, in the West Bank, 286 assaults on healthcare have impeded the delivery of care, including the distribution of vital pharmaceuticals and equipment, hospital closures, and denial of ambulance access (25).

This prevailing political instability and recurrent episodes of violence have engendered a dangerous environment for both patients and healthcare professionals. The difficulties in delivering and accessing healthcare services are made even more complex by restricted mobility, limited resources, and persistent security concerns (26). Nevertheless, there is a lack of research that explicitly focuses on work burnout in the Palestinian healthcare system during periods of political conflict (27). Only a few studies have been conducted among health professionals, and none have specifically focused on the current war period or examined coping strategies (28, 29). This research aimed to examine burnout and coping strategies among healthcare workers in the West Bank who provided medical care to Palestinians during the political crisis that started on October 7, 2023. Also, the study aimed to assess the relationship between burnout, coping strategies, and sociodemographic factors. Finally, the study looked at the factors that might predict the development of burnout symptoms among healthcare professionals.

## Methods

### Study design and sampling

The study was a descriptive cross-sectional survey that ran from 15 January 2024 to 1 February 2024. An online sample size calculator^1^ determined a sample size of 587, with a 5% margin of error and a 95% confidence interval, supposing that 50% of healthcare teams experience burnout. The study targeted all Palestinian health professionals currently working in the West Bank and Jerusalem during the ongoing Gaza war and political violence, including doctors, nurses, pharmacists, and allied professionals (e.g., anesthetics technicians, X-ray technicians, and medical laboratory). Health professionals in Gaza were unable to fill out the questionnaire because of the catastrophic war conditions, ongoing displacement, and restricted Internet access. Participants were chosen using convenience and snowball sampling methods. Data were gathered using an anonymous online self-administered survey. In response to Israeli military restrictions on mobility and closures in the West Bank and Jerusalem, participants were asked to complete an electronic version of the questionnaire, designed using Google Forms. The study link was distributed to participants via a variety of channels, including social media, WhatsApp, emails, and work-related organization websites. Furthermore, participants were asked to share the link with mental health practitioners throughout the country, and 713 from Jerusalem and the West Bank responded.

### Tools and measures

The study used a self-reported questionnaire, which had the following three sections:

*Section one included a socio-demographic sheet* to collect information related to the participants’ age, gender, living place, marital status, occupation, work place, education level, monthly income, governorate (north governorate included Nablus, Jenin, Qalqilia, Tulkarim, Tubas, and Salfeet, middle governorate included Ramallah, East Jerusalem, and Jericho, and south governorate included Hebron and Bethlehem), years of experience and working hours per day.

*The second section had the shortened version of the Maslach Burnout Questionnaire* (9) which comprises 9 items that evaluate three subscales: personal accomplishment (PA) (3 items), depersonalization (DP) (3 items), and emotional exhaustion (EE) (3 items). The score of each subscale could range from minimum 0 to maximum 18. High score of EE and DP and a lower score of PA indicates a higher level of burnout. For EE and DP, subscale score of 0–9 was categorized as “no to low burnout” and subscale score of 10–18 was regarded as “moderate to severe burnout.” It was the opposite for PA because higher PA scores indicate lesser burnout. A seven-point Likert scale, with 0 representing “Never” and 6 representing “Every day,” can be used to answer questions.

Validated burnout thresholds for the a-MBI were employed (>6 for depersonalization, >9 for emotional exhaustion, and < 9 for personal achievement). In accordance with prior research, participants were classified as experiencing burnout if they exceeded the established threshold scores in either the depersonalization or emotional exhaustion domains (30, 31). Depersonalization and emotional exhaustion subscales had respective Cronbach’s alphas of 0.82 and 0.77 and Personal accomplishment 0.71.

*The third section had the Brief COPE scale* which was developed by Carver (32) and consisted of 28 questions. Both cognitive and behavioral strategies of coping are included and for each category, respondents indicate whether they have used a coping response on a four-point Likert scale (1 = I have not been doing this at all, 2 = I have been doing this a little bit; 3 = I have been doing this an average amount; 4 = I have been doing this a lot) and the higher score represents greater coping strategies used by the respondents. The Brief COPE scale assesses the following coping mechanisms: self-distraction, active coping, denial, substance use, emotional support, instrumental support, behavioral disengagement, venting, positive reframing, planning, humor, acceptance, religion, and self-blame. The internal consistency coefficient (Cronbach’s *α*) was 0.850.

The survey was translated into Arabic and back into English. The Arabic terminology was piloted by 20 health professionals and reviewed by five experts to verify accuracy and understandability.

### Data analysis

The data were analyzed using SPSS version 25 (IBM Corp., Chicago, Illinois, USA). A descriptive analysis was performed using the means and the standard deviations for the quantitative variables, and frequencies and percentages for the categorical variables. Chi-squared test, Student’s *t*-test, Mann–Whitney, and Analysis of variance were used to analyze the relationships between the sociodemographic, occupational, work condition, coping strategies, and MBI dimensions. Statistically significant variables were further analyzed with multivariate logistic regression. A *p* < 0.05 was considered sufficient for statistical significance. Adjusted odds ratio and 95% confidence interval were also reported.

### Ethical approval and consent to participate

The Declaration of Helsinki was followed in the implementation of all study methods. Al Quds University Research Ethical Committee approval was obtained (Ref No: 347/REC/2023). There was anonymity in this online survey. At the outset of the survey, written information was given regarding its goal and the intended use of the data. By completing the questionnaire, the participants gave their informed consent to take part in the research.

## Results

### Study participant’s socio-demographic characteristics

The sample included 713 healthcare professionals, 60.3% of whom were female. 53.2% were aged 18–30, 43% were unmarried, and 47.3% earned more than $1,150 every month. Nursing accounted for 57.2% of participants, with 64.7% working in government hospitals and primary care centers. Additionally, 70% had a bachelor’s degree, 46.1% had been working in their present position for more than 6 years, and 64.9% worked an eight-hour shift daily as seen in Table 1.

**Table 1:** Sociodemographic characteristics occupation and work conditions of study participants.

		Frequency (N)	Percent (%)
Gender	Male	283	39.7%
	Female	430	60.3%
Age (years)	18–30	379	53.2%
	31–40	191	26.8%
	41+	143	20.1%
Living place	City	341	47.8%
	Village	331	46.4%
	Refugee camp	41	5.8%
Marital status	Single	308	43.2%
	Not single*	405	56.8%
Monthly income (US$)	No income	50	7.0%
	< 570	52	7.3%
	570–1,150	275	38.6%
	1,151–1700	209	29.3%
	1701+	127	17.8%
Occupation	Allied profession	134	18.8%
	Physicians	111	15.6%
	Pharmacists	60	8.4%
	Nurses	408	57.2%
Work place	Governmental	461	64.7%
	Private	172	24.1%
	Others**	80	11.2%
Governate	North	230	32.3%
	Middle	221	31.0%
	South	262	36.7%
Education level	Bachelor	504	70.7%
	Master	98	13.7%
	Doctoral	23	3.2%
	Diploma	88	12.3%
Years of experience	< 1	147	20.6%
	1–3	120	16.8%
	4–6	117	16.4%
	7–10	90	12.6%
	11–15	112	15.7%
	16+	127	17.8%
Working hours per day	8	463	64.9%
	9–12	212	29.7%
	13–16	25	3.5%
	17+	13	1.8%

*Married/widowed/divorced. ** Non-governmental organizations, civil institutes, and international agencies.

### Prevalence of burnout

The overall burnout mean was 22.47 (SD 8.65), with a median of 22 (25th percentile = 17, 75th percentile = 28). Thus, 24% had a score less than 17 (*n* = 171), 3.1% had a score between 18 and 28 (*n* = 22), and 72.9% had a high score more than 28 (*n* = 713). According to the burnout subscale analysis, the prevalence of emotional exhaustion in the sample was 44.2% (*n* = 315), 9.8% (*n* = 70) for depersonalization, and 72.2% (*n* = 515) had poor personal accomplishment. For nurses, 40.9% had emotional exhaustion, 9.8% had depersonalization and 71.8% had poor personal accomplishment. For physicians, 56.8% had emotional exhaustion, 10.8% had depersonalization and 78.4% % had poor personal accomplishment.

### Coping strategies subscale

Table 2 shows the description of the coping strategies subscale. Religion showed the highest mean 6.45 (SD ± 1.59) and the highest 75th percentile (8) and substance abuse showed the lowest mean 2.45 (SD ±1.18) and the lowest 75th percentile (2).

**Table 2:** Coping strategies subscale.

	Mean (SD)		Percentile 25	Median	Percentile 75
Active coping	5.49	(±1.52)	4.00	6.00	7.00
Use of informational support	4.35	(±1.50)	3.00	4.00	5.00
Positive reframing	4.44	(±1.72)	3.00	4.00	6.00
Planning	4.95	(±1.56)	4.00	5.00	6.00
Emotional support	4.05	(±1.69)	3.00	4.00	5.00
Venting	4.63	(±1.50)	4.00	5.00	6.00
Humor	2.70	(±1.29)	2.00	2.00	3.00
Acceptance	5.71	(±1.60)	5.00	6.00	7.00
Religion	6.45	(±1.59)	5.00	7.00	8.00
Self-blame	3.80	(±1.54)	3.00	4.00	5.00
Self-distraction	4.93	(±1.51)	4.00	5.00	6.00
Denial	4.32	(±1.80)	3.00	4.00	6.00
Substance use	2.45	(±1.18)	2.00	2.00	2.00
Behavioral disengagement	3.78	(±1.50)	2.00	4.00	5.00

SD, Standard Deviation.

### Association between burnout subscale and participant’s socio-demographic characteristics

Subgroup analysis was conducted to explore the burnout level in different socio-demographics (Table 3). The findings showed that emotional exhaustion subscale was significantly associated with participants’ age (*p* = 0.005), monthly income (*p* = 0.023), education level (*p* = 0.002), occupation (*p* = 0.004), years of experience (*p* < 0.001), and the number of daily working hours (*p* = 0.002). Also, depersonalization subscale was associated with participants’ gender (*p* = 0.009), age (*p* < 0.001), marital status (*p* = 0.003), monthly income (*p* = 0.035), place of work (*p* = 0.048), the number of years of experience (*p* = 0.001), and the number of daily working hours (*p* = 0.009). In addition, personal accomplishment subscale was associated with participants’ gender (*p* = 0.022), education level (*p* = 0.003), monthly income (*p* = 0.030), marital status (*p* = 0.015), place of work (*p* = 0.026), and the number of years of experience (*p* < 0.001).

**Table 3:** Associations between socio-demographic, occupation, and work condition variables and Burnout symptom level (*N* = 715).

Socio-demographic variables		Emotional exhaustion					Depersonalization					Personal accomplishment				
		≤9 low to moderate *N* = 298		>9 high *N* = 315		Chi-squared	≤6 low to moderate *N* = 298		>6 high *N* = 315		chi-squared	>9 low to moderate *N* = 515		≤9 high *N* = 198		Chi-squared
		N	%	N	%	*P*-value	N	%	N	%	*P*-value	N	%	N	%	*P*-value
Occupation	Allied profession	82	20.6%	52	16.5%*	0.004	122	19.0%	12	17.1%	0.971	95	18.4%	39	19.7%	0.366
	Physicians	48	12.1%	63	20.0%		99	15.4%	12	17.1%		87	16.9%	24	12.1%	
	Pharmacists	27	6.8%	33	10.5%		54	8.4%	6	8.6%		40	7.8%	20	10.1%	
	Nurses	241	60.6%	167	53.0%		368	57.2%	40	57.1%		293	56.9%	115	58.1%	
Work place	Government	252	63.3%	209	66.3%	0.136	421	65.5%	40	57.1%	0.048	344	66.8%	117	59.1%*	0.026
	Private	93	23.4%	79	25.1%		156	24.3%	16	22.9%		123	23.9%	49	24.7%	
	Others	53	13.3%	27	8.6%		66	10.3%	14	20.0%		48	9.3%	32	16.2%	
Gender	Male	156	39.2%	127	40.3%	0.761	245	38.1%	38	54.3%	0.009^*^	191	37.1%	92	46.5%*	0.022
	Female	242	60.8%	188	59.7%		398	61.9%	32	45.7%		324	62.9%	106	53.5%	
Age (years)	18–30	221	55.5%	158	50.2%*	0.005	330	51.3%	49	70.0%	0.000^*^	253	49.1%	126	63.6%*	0.000
	31–40	88	22.1%	103	32.7%		171	26.6%	20	28.6%		139	27.0%	52	26.3%	
	41+	89	22.4%	54	17.1%		142	22.1%	1	1.4%		123	23.9%	20	10.1%	
Living	City	176	44.2%	165	52.4%	0.094	302	47.0%	39	55.7%	0.154	249	48.3%	92	46.5%	0.744
	Village	197	49.5%	134	42.5%		301	46.8%	30	42.9%		235	45.6%	96	48.5%	
	Refugee camp	25	6.3%	16	5.1%		40	6.2%	1	1.4%		31	6.0%	10	5.1%	
Marital status	Single	182	45.7%	126	40.0%	0.125	266	41.4%	42	60.0%	0.003	208	40.4%	100	50.5%	0.015
	Not single	216	54.3%	189	60.0%		377	58.6%	28	40.0%		307	59.6%	98	49.5%	
Monthly Income (US $)	No income	37	9.3%	13	4.1%*	0.023	43	6.7%	7	10.0%	0.035	31	6.0%	19	9.6%*	0.030
	< 570	35	8.8%	17	5.4%		49	7.6%	3	4.3%		37	7.2%	15	7.6%	
	570–1,150	144	36.2%	131	41.6%		239	37.2%	36	51.4%		186	36.1%	89	44.9%	
	1,150–1700	115	28.9%	94	29.8%		190	29.5%	19	27.1%		164	31.8%	45	22.7%	
	1701+	67	16.8%	60	19.0%		122	19.0%	5	7.1%		97	18.8%	30	15.2%	
Area of work	North	119	29.9%	111	35.2%	0.128	212	33.0%	18	25.7%	0.436	175	34.0%	55	27.8%	0.248
	Middle	135	33.9%	86	27.3%		196	30.5%	25	35.7%		153	29.7%	68	34.3%	
	South	144	36.2%	118	37.5%		235	36.5%	27	38.6%		187	36.3%	75	37.9%	
Degree level	Bachelor	280	70.4%	224	71.1%*	0.002	448	69.7%	56	80.0%	0.074	344	66.8%	160	80.8%*	0.003
	Master	46	11.6%	52	16.5%		89	13.8%	9	12.9%		81	15.7%	17	8.6%	
	Doctoral	9	2.3%	14	4.4%		20	3.1%	3	4.3%		20	3.9%	3	1.5%	
	Diploma	63	15.8%	25	7.9%		86	13.4%	2	2.9%		70	13.6%	18	9.1%	
Years of experience	< 1	106	26.6%	41	13.0%*	0.000	126	19.6%	21	30.0%	0.001	93	18.1%	54	27.3%*	0.000
	1–3	59	14.8%	61	19.4%		108	16.8%	12	17.1%		84	16.3%	36	18.2%	
	4–6	61	15.3%	56	17.8%		101	15.7%	16	22.9%		84	16.3%	33	16.7%	
	7–10	35	8.8%	55	17.5%		77	12.0%	13	18.6%		55	10.7%	35	17.7%	
	11–15	56	14.1%	56	17.8%		105	16.3%	7	10.0%		91	17.7%	21	10.6%	
	16+	81	20.4%	46	14.6%		126	19.6%	1	1.4%		108	21.0%	19	9.6%	
Working hours per day	8	280	70.4%	183	58.1%*	0.002	427	66.4%	36	51.4%	0.009	334	64.9%	129	65.2%	0.517
	9–12	103	25.9%	109	34.6%		184	28.6%	28	40.0%		157	30.5%	55	27.8%	
	13–16	12	3.0%	13	4.1%		23	3.6%	2	2.9%		15	2.9%	10	5.1%	
	17+	3	0.8%	10	3.2%		9	1.4%	4	5.7%		9	1.7%	4	2.0%	

### Burnout and coping strategies

Subgroup analysis was conducted to explore the burnout level in different coping strategies (Table 4). Emotional Exhaustion subscale was significantly associated with active coping (*p* = 0.013), emotional support (*p* = 0.046), venting (*p* = 0.042), self-blame (*p* = 0.10), and behavioral disengagement (*p* = 0.001). Also, depersonalization subscale was significantly associated with active coping (*p* = 0.015), humor (*p* = <0.001), religion (*p* = 0.004), self-blame (*p* = 0.001), denial (*p* = 0.018), substance use (*p* = <0.001), and behavioral disengagement (*p* = <0.001). In addition, the personal accomplishment subscale was significantly associated with active coping (*p* = 0.002), positive reframing (0.03), humor (<0.001), self-blame (0.006), acceptance (0.03), religion (0.001), denial (0.037), substance use (<0.001), and behavioral disengagement (0.015).

**Table 4:** Association between brief coping and Burnout symptom level (*N* = 715).

	Emotional exhaustion					Depersonalization					Personal accomplishment				
	≤9 low to moderate *N* = 298		>9 high *N* = 315		T-test	≤6 low to moderate *N* = 677		>6 high *N* = 36		T-test	>9 low to moderate *N* = 515		≤9 high *N* = 198		*T*-test
	Mean	SD	Mean	SD	Sig.	Mean	SD	Mean	SD	Sig.	Mean	SD	Mean	SD	Sig.
Active coping	5.62	1.51	5.33	1.53	**0.013**	5.54	1.54	5.07	1.29	**0.015**	5.60	1.51	5.21	1.51	**0.002**
Use of informational support	4.29	1.45	4.42	1.56	0.323	4.31	1.50	4.70	1.46	**0.038**	4.34	1.47	4.36	1.58	0.920
Positive reframing	4.50	1.69	4.36	1.76	0.267	4.40	1.74	4.73	1.52	0.135	4.36	1.74	4.65	1.65	**0.030**
Planning, items	4.96	1.57	4.94	1.54	0.845	4.95	1.57	4.94	1.44	0.976	4.99	1.57	4.83	1.53	0.259
Emotional support	4.17	1.73	3.90	1.63	**0.046**	4.02	1.71	4.29	1.42	0.212	3.99	1.68	4.20	1.71	0.113
Venting	4.53	1.53	4.76	1.45	**0.042**	4.59	1.51	4.93	1.37	0.076	4.67	1.49	4.53	1.51	0.236
Humor	2.69	1.33	2.71	1.24	0.358*	2.61	1.21	3.47	1.68	0.000*	2.56	1.10	3.07	1.62	0.000*
Acceptance	5.73	1.58	5.67	1.61	0.619	5.73	1.61	5.44	1.43	0.147	5.78	1.63	5.52	1.50	**0.030**
Religion	6.52	1.56	6.38	1.62	0.275	6.51	1.56	5.94	1.70	**0.004**	6.58	1.53	6.13	1.67	**0.001**
Self-blame	3.68	1.53	3.96	1.55	**0.010**	3.70	1.50	4.73	1.62	**0.000**	3.70	1.51	4.06	1.60	**0.006**
Self-distraction	4.98	1.52	4.85	1.50	0.353	4.91	1.53	5.03	1.38	0.550	4.88	1.50	5.06	1.56	0.147
Denial	4.21	1.81	4.45	1.78	0.053	4.26	1.80	4.80	1.72	**0.018**	4.24	1.81	4.52	1.75	**0.037**
Substance use	2.51	1.23	2.38	1.12	0.054*	2.37	1.07	3.24	1.74	0.000*	2.28	0.94	2.91	1.57	0.000*
Behavioral disengagement	3.63	1.49	3.98	1.49	**0.001**	3.71	1.49	4.46	1.38	**0.000**	3.70	1.47	4.01	1.55	**0.015**

SD, Standard deviation; Sig., Significance. *Mann–Whitney test significance.

### Multivariate logistic regression model

Multivariate analysis supported some of the previously stated findings. The results revealed that increasing participants’ years of experience, working hours per day, and usage of behavioral disengagement increased the likelihood of emotional exhaustion (AOR = 1.26, 95% CI = 1.21–1.461). Additionally, active coping, substance use, informational support, and emotional support reduced the likelihood of experiencing emotional exhaustion. Furthermore, active coping reduced the probability of experiencing depersonalization (AOR = 0.745, 95% CI = 0.656–0.938), but substance use and self-blame increased the likelihood of having it. Furthermore, increasing the number of experience years and using active coping reduced the likelihood of having personal accomplishment (AOR = 0.846, 95%CI = 0.755–0.948), whereas substance use increased the odds of having it (AOR = 1.457, 95%CI = 1.271–1.670) (Table 5).

**Table 5:** Multivariate regression analysis for determinants of Burnout symptom.

	Emotional exhaustion				Depersonalization				Personal accomplishment			
	Sig.	AOR	95% CI AOR				95% CI AOR				95% CI AOR	
			Lower	Upper	Sig.	AOR	Lower	Upper	Sig.	AOR	Lower	Upper
Age (years)					0.028							
**18–30**						Ref.						
**31–40**					0.671	0.883	0.496	1.570				
**41+**					**0.008**	0.066	0.009	0.487				
Years of work												
> 1 year		Ref.										
1–3 years	**0.002**	2.279	1.343	3.867					0.546	0.848	0.495	1.450
4–6 years	**0.009**	2.033	1.189	3.476					0.282	0.742	0.430	1.278
7–10 years	**0.000**	3.476	1.938	6.234					0.335	1.320	0.751	2.319
11–15 years	**0.002**	2.344	1.363	4.030					**0.015**	0.475	0.261	0.868
16+ years	0.286	1.339	0.783	2.288					**0.003**	0.390	0.212	0.719
Hours you work per day												
8 h		Ref.										
9–12 h	0.087	1.359	0.957	1.931								
13–16 h	0.454	1.381	0.593	3.216								
17+ hours	**0.023**	4.923	1.249	19.406								
Coping strategies												
Active coping	**0.015**	0.875	0.785	0.975	**0.008**	0.784	0.656	0.938	**0.004**	0.846	0.755	0.948
Substance use	**0.016**	0.830	0.713	0.966	**0.005**	1.276	1.077	1.512	**0.000**	1.457	1.271	1.670
Self Balme					**0.001**	1.361	1.141	1.624				
Use of informational support	**0.004**	1.225	1.067	1.406								
Emotional support	**0.002**	0.815	0.718	0.925								
Behavioral disengagement	**0.000**	1.260	1.121	1.416								

AOR, Adjusted Odds Ratio; 95% CI, 95% confidence interval. Values in bold are significant.

## Discussion

The mental health of health professionals is a critical problem because they are in charge of delivering long-term care to the people. The current study found that more than two-thirds of Palestinian health professionals (72.9%) experienced burnout, 44.2% had emotional exhaustion, 9.8% had depersonalization, and 72.2% experienced low personal accomplishment. Burnout findings are considered high when compared to other research in the literature review. Hamdan and Hamra (28) conducted study in the West Bank and Gaza to assess burnout levels among healthcare professionals (nurses, physicians, and administrative staff) in hospital emergency rooms during non-war periods, it was found that among emergency department staff, 64.0% experienced significant emotional exhaustion, 38.1% experienced depersonalization, and 34.6% had poor personal achievement (28). In Uganda, Kabunga et al. reported that 39.8% of respondents had significant levels of burnout in none war-times (33). In Indonesia, one research in none war-times revealed that 15.5% had emotional exhaustion (EE), 5.2% had depersonalization (DP), and 39.2% had personal accomplishment (34). In a cross-sectional research in Ukraine in war-times, EE and DP were found in 31.6 and 33.4% of physicians, respectively, as well as 17.9 and 18.2% of nurses (15). Another study found that 67% of Polish physicians suffered from burnout in non-war times (35).

Other research, on the other hand, found high level of burnout similar to the current study. According to one systematic review, the prevalence of high burnout varied from 0.9 to 40%, high emotional exhaustion from 0 to 49.7%, depersonalization from 0 to 59.6%, and low personal accomplishment from 0 to 60% (36). According to Alhaffar et al.’s study, during the Syrian conflict, 77.9 and 54.6% of physicians reported significant levels of EE and DP (14) and Elhadi et al. found that 67.1% of Libyan physicians had high EE and 47.4% had high DP after civil war (12). These findings may indicate that political conflict and wars raise burnout level among health professionals. Therefore, paying attention to their needs and their psychological status is very crucial in wartime.

The findings of the current study revealed several factors that may lead to an increase in burnout among Palestinian health professionals. The study found that those who worked more than 16 h per day and health professionals with 7–10 years of work experience were at the greatest risk of developing emotional exhaustion, followed by those with 1–3 years of experience. Also, those with 4–6 years of experience were at a higher risk of emotional exhaustion than health professionals with less than 1 year of experience.

These findings are supported by other studies in the literature review (37–39). Long years of employment can be related to people gradually taking on increasingly major responsibilities, which causes increased stress. Furthermore, professionals may use fewer coping strategies at work and experience chronic fatigue as a result of repeatedly experiencing the same issues day after day for a long period (40). In Palestine, with the start of the continuing battle in Gaza on October 7th, the majority of Palestinian hospital facilities declared an emergency. They directed all healthcare staff to postpone their holidays and return to work to meet the urgent situation. Also, due to the Israeli military’s blockade of Palestinian cities and implementation of mobility restrictions in the West Bank, a large number of these healthcare professionals were asked to remain at their jobs to solve the staffing shortage. As a result, health professionals who work long hours and have prolonged careers are more prone to develop emotional exhaustion in conflict situations. Long working hours are an indication of concern since they can lead to the onset of medical problems. A high level of burnout can lead to stress-related diseases and mental problems such as depression, anxiety, and low self-esteem. It can also lead to an increase in the occurrence of physical health issues such as sleep disruptions, migraines, cardiovascular disease, and other diseases (41).

On the other hand, our study found that professionals with more than a decade of work experience were more likely to have personal accomplishments and less likely to experience burnout than those with less than a year of experience. Work experience can be linked to workers’ capacity and autonomy in shaping their responsibilities positively. Employees who deliberately created a productive and stimulating work environment for themselves reported an increase in psychological resources such as hope, resilience, self-efficacy, and optimism, as well as improved job satisfaction (42). Habibisaravi et al. found that working professionals with extensive work experience showed stronger resilience and were capable of efficiently balancing their personal lives with their professional obligations lowering their burnout levels (43). Thus, our findings highlight the need for early screening for mental and physical health problems, such as cardiovascular disease, among healthcare professionals. Additionally, it emphasizes the need to limit working hours to a maximum of 40 per week.

Furthermore, the current study found that Palestinian health professionals used several coping strategies to minimize burnout. Active coping, substance use, informational support, and emotional support all decreased the probability of experiencing emotional exhaustion. Furthermore, active coping decreased the likelihood of developing depersonalization, but substance use increased personal accomplishment. Other research revealed similar findings. Menaldi et al. found that active coping, instrumental support, and substance use reduced the likelihood of experiencing EE (34). Doolittle and Windish showed that active coping was highly related to decreased emotional exhaustion and depersonalization. Problem-focused coping strategies, such as active coping and informational support, are active attempts to deal with a stressful situation by engaging in problem-solving activities to modify the situation or explore alternatives (44). Positive emotional coping strategies, such as emotional support, involve demonstrating compassion and understanding to oneself while attempting to address a problem independently, regardless of the outcome. These strategies also include making cognitive modifications that help produce positive feelings and enhance a sense of calmness in difficult circumstances, hence reducing EE and depersonalization (45). Furthermore, our data indicated that active coping had a negative relationship with personal accomplishment. According to Doolittle and Windish et al., personal accomplishment refers to the things that support and add value to the everyday tasks of patient care, therefore influencing one’s achievement. However, using active coping strategies alone may not be enough to alleviate the severe physical demands and reflexive dehumanization that come with all of the complicated aspects of patient care in challenging circumstances in areas of conflict (44).

Interestingly, our research indicated that substance use decreased EE and enhanced personal accomplishment. Avoidant coping strategies, such as substance use, are not necessarily maladaptive and can have different impacts depending on the level of stress (46, 47). Cecil et al. revealed a substantial negative relationship between burnout syndrome and alcohol use. They proposed that the frequency of alcohol intake in individuals might be more strongly related to the joy of drinking and socializing, lowering their level of burnout and EE and improving their relationships with others and their patients (48). Thus, substance use may boost professionals’ confidence and belief in their capacity to manage difficult situations, resulting in a more favorable appraisal of their accomplishments and personal fulfillment. However, the current study found that substance use increased the likelihood of depersonalization. Depersonalization (DP) is characterized by a lack of empathy and a cold, detached manner (49). Elkardi et al. showed that burnout can lead to substance use among healthcare professionals (50). Individuals who are undergoing burnout may use drugs as a coping strategy. The use of these substances constitutes an emotional confrontation since they aim to interfere with the individual’s ability to understand challenging situations through an escape strategy, which may result in depersonalization (48). Some studies indicate that the use of substances might provide a rapid but temporary relief response (51, 52). Therefore, this coping method may provide temporary relief from burnout symptoms, but it has the potential to lead to serious problems such as drug addiction. A study revealed that physicians who used alcohol four or more times weekly had a 3.3-fold greater chance of acquiring burnout syndrome (53). Other research, however, found no significant association between substance use and burnout (54). It is worth noting that the Brief Cope Scale does not indicate the sort of substance used, such as smoking, alcohol, or narcotics. As a result, more research is needed to determine what substances Palestinian health professionals take to cope with the stress of political violence.

Furthermore, the current study’s findings revealed that behavioral disengagement increased the probability of experiencing emotional exhaustion, whereas self-blame raised the likelihood of depersonalization. Other research found similar findings (44, 55). According to Leo et al., some escapist-avoidance strategies, such as disengagement, that lack a problem-solving approach may impede one’s ability to cope with a situation using their own or external resources, resulting in emotional exhaustion (13). Furthermore, persons who engage in self-blame are often seen to be competent in their domains of expertise. Individuals with a high sense of diligence may accept responsibility for work that others may not see as their own. This behavior makes them more likely to develop stress-related disorders and burnout (56).

This research has limitations. Convenience sampling and cross-sectional designs reduce the ability to demonstrate causal relationships and affect the representativeness of the sample and the generalizability of the findings. Furthermore, there is a possibility of reporting bias due to the use of a self-reported questionnaire. Given that recruiting took place using platforms such as Google Docs and WhatsApp, it is probable that health professionals already employed in the impacted areas, including the Gaza Strip, do not have access to or the chance to use this technology. As a result, this situation may have an impact on the sample’s representativeness. Additionally, few studies examine burnout among healthcare professionals during wartime. As a result, comparing our findings to other studies is limited. Also, in this study we were not able to calculate the response rate due to the use of anonymous online questionnaire. Despite these limitations, the findings of the current study provide light on the psychological well-being of health workers living in conflict-affected countries. This study provides a major contribution to the current literature since it is the first to analyze burnout and coping methods among Palestinian healthcare professionals throughout periods of armed conflict and political violence.

### Implication for practice

As a result, professionals must develop effective coping strategies early in their careers to reduce the risk of developing burn out. Training sessions or seminars can help health practitioners understand their typical defense style and how it affects them and their ability to provide treatment during political violence. Furthermore, excessive workloads, prolonged working hours, a stressful work environment, and substandard workplace conditions have been identified as risk factors for stress, maladaptation, and burnout among health professionals. Consequently, health policymakers and managers can enhance stress adaptation among these health professionals during wartime by improving sleep quality and hours, offering a nutritious diet, reducing excessive workloads, promoting regular physical exercise and relaxation techniques, facilitating social engagement, ensuring a suitable work-life balance, and guaranteeing the physical safety of their employees (57). Further, health professionals should receive psychological support, treatment, and psychotherapy at the workplace to overcome their negative emotions to improve their well-being and a sense of efficacy and competence. Additionally, the study findings may underscore the necessity of implementing regulations and policies that safeguard and assist healthcare professionals during conflict and war. Thus, our research suggests the need for early screening for medical and mental problems, such as cardiovascular disease, among healthcare workers. Additionally, it highlights the need to limit working hours to a maximum of 40 per week.

More qualitative and quantitative research is required to fully understand the factors that increase burn out among health professionals and coping strategies for dealing with patients during political violence. Additional study is required to examine which drugs Palestinian health professionals use to deal with the stress of political violence. Finally, further research is needed to assess burnout levels in certain health professions in order to investigate burnout levels and related causes during wars and political violence.

## Conclusion

The study found that burnout symptoms are common among Palestinian health professionals, particularly in less experienced professionals and those who work for long ours. In addition, using self-blame, and behavioral disengagement as coping mechanisms increases the likelihood of developing burnout symptoms. The study highlights the significance of identifying stressors faced by health professionals during wartime and implementing measures to prevent occupational burnout. Consequently, health professionals need immediate assistance in enhancing their mental wellbeing through psychological intervention and, and comprehensive training in stress management.

## Data Availability

The original contributions presented in the study are included in the article/supplementary material, further inquiries can be directed to the corresponding author.

## Footnotes

^1^ https://www.calculator.net/sample-size

## References

[1] Solis G. (2016). The law of armed conflict: international humanitarian law in war. Cambridge University Press.

[2] Garry S Checchi F. Armed conflict and public health: into the 21st century 2020; 42(3): e287-e298. doi: 10.1093/pubmed/fdz095. Erratum in. J Public Health. (2021) 43:e110. doi: 10.1093/pubmed/fdaa036, PMID: 32300804

[3] Rizzi D Ciuffo G Sandoli G Mangiagalli M de Angelis P Scavuzzo G. Running away from the war in Ukraine: the impact on mental health of internally displaced persons (IDPs) and refugees in transit in Poland. Int J Environ Res Public Health. (2022) 19:16439. doi: 10.3390/ijerph192416439, PMID: 36554321 PMC9778520

[4] AlKhaldi M Kaloti R Shella D Al Basuoni A Meghari H. Health system's response to the COVID-19 pandemic in conflict settings: policy reflections from Palestine. Glob Public Health. (2020) 15:1244–56. doi: 10.1080/17441692.2020.1781914, PMID: 32552389

[5] Narwal S Jain S. Building resilient health systems: patient safety during COVID-19 and lessons for the future. J Health Manag. (2021) 23:166–81. doi: 10.1177/0972063421994935

[6] Abed Alah M. Echoes of conflict: the enduring mental health struggle of Gaza’s healthcare workers. Confl Heal. (2024) 18:21. doi: 10.1186/s13031-024-00577-6, PMID: 38481328 PMC10938685

[7] Dominic C Gopal D Sidhu A. ‘It’s like juggling fire daily’: well-being, workload and burnout in the British NHS - a survey of 721 physicians. Work. (2021) 70:395–403. doi: 10.3233/WOR-205337, PMID: 34633337

[8] De Hert S. Burnout in healthcare workers: prevalence, impact and preventative strategies. Local Reg Anesth. (2020) 13:171–83. doi: 10.2147/LRA.S240564, PMID: 33149664 PMC7604257

[9] Maslach C Jackson SE Leiter MP. Maslach burnout inventory. Palo Alto, CA: Consulting Psychologists Press (1996).

[10] Nápoles J. Burnout: a review of the literature. Update: applications of research in music. Education. (2021) 40:875512332110376. doi: 10.1177/87551233211037669

[11] Harrell M Selvaraj SA Edgar M. DANGER! Crisis health Workers at Risk. Int J Environ Res Public Health. (2020) 17:5270. doi: 10.3390/ijerph17155270 32707800 PMC7432711

[12] Elhadi M Khaled A Malek AB El-Azhari AE Gwea AZ Zaid A. Prevalence of anxiety and depressive symptoms among emergency physicians in Libya after civil war: a cross-sectional study. BMJ Open. (2020) 10:e039382. doi: 10.1136/bmjopen-2020-039382, PMID: 32859667 PMC7454180

[13] Leo CG Sabina S Tumolo MR Bodini A Ponzini G Sabato E. Burnout among healthcare workers in the COVID 19 era: a review of the existing literature. Front Public Health. (2021) 9:750529. doi: 10.3389/fpubh.2021.750529, PMID: 34778184 PMC8585922

[14] Alhaffar BA Abbas G Alhaffar AA. The prevalence of burnout syndrome among resident physicians in Syria. J Occup Med Toxicol. (2019) 14:31. doi: 10.1186/s12995-019-0250-0 31827575 PMC6902600

[15] Owoc J Mańczak M Olszewski R Shevchuk O Zaporozhan S Korda M. (2023). Can war help in better understanding of burnout phenomenon? A cross sectional study of burnout in Ukrainian medical staff. Available at: https://ssrn.com/abstract=4757684 (Accessed June, 12, 2024).

[16] Lazarus RS. From psychological stress to the emotions: a history of changing outlooks. Annu Rev Psychol. (1993) 44:1–22. doi: 10.1146/annurev.ps.44.020193.000245 8434890

[17] Fitzgibbon K Murphy KD. Coping strategies of healthcare professional students for stress incurred during their studies: a literature review. J Ment Health. (2023) 32:492–503. doi: 10.1080/09638237.2021.2022616, PMID: 35020566

[18] Theodoratou M Farmakopoulou I Kougioumtzis G Kaltsouda A Siouti Z Sofologi M. Emotion-focused coping, social support and active coping among university students: gender differences. J Psychol Clin Psychiatry. (2023) 14:5–9. doi: 10.15406/jpcpy.2023.14.00720

[19] Ding Y Fu X Liu R Hwang J Hong W Wang J. The impact of different coping styles on psychological distress during the COVID-19: the mediating role of perceived stress. Int J Environ Res Public Health. (2021) 18:10947. doi: 10.3390/ijerph182010947, PMID: 34682693 PMC8535725

[20] Kua Z Hamzah F Tan PT Ong LJ Tan B Huang Z. Physical activity levels and mental health burden of healthcare workers during COVID-19 lockdown. Stress Health. (2022) 38:171–9. doi: 10.1002/smi.3078, PMID: 34231968 PMC8420337

[21] Alameddine M Fouad FM Diaconu K Jamal Z Lough G Witter S. Resilience capacities of health systems: accommodating the needs of Palestinian refugees from Syria. Soc Sci Med. (2019) 220:22–30. doi: 10.1016/j.socscimed.2018.10.018, PMID: 30390471

[22] PNIPH. (2018). Overview of public health in Palestine. Available at: https://www.pniph.org/en/about/overview-of-public-health-in-palestine (Accessed November, 2024).

[23] Ben Saad H Dergaa I. Public health in peril: assessing the impact of ongoing conflict in Gaza strip (Palestine) and advocating immediate action to halt atrocities. New Asian. J Med. (2023) 1:1–6. doi: 10.61838/kman.najm.1.2.1

[24] Søvold LE Naslund JA Kousoulis AA Saxena S Qoronfleh MW Grobler C. Prioritizing the mental health and well-being of healthcare workers: an urgent global public health priority. Front Public Health. (2021) 9:679397. doi: 10.3389/fpubh.2021.679397, PMID: 34026720 PMC8137852

[25] Palestinian Central Bureau of Statistics (2024). The Palestinian central Bureau of Statistics “PCBS” issues a press release on world health day. Available at: https://www.pcbs.gov.ps/portals/_pcbs/PressRelease/Press_En_WorldHealthDay2024E.pdf (Accessed November, 2024).

[26] Jabali O Ayyoub AA Jabali S. Navigating health challenges: the interplay between occupation-imposed movement restrictions, healthcare access, and community resilience. BMC Public Health. (2024) 24:1297. doi: 10.1186/s12889-024-18817-y, PMID: 38741152 PMC11089674

[27] Shawahna R Maqboul I Ahmad O Al-Issawy A Abed B. Prevalence of burnout syndrome among unmatched trainees and residents in surgical and nonsurgical specialties: a cross-sectional study from different training centers in Palestine. BMC Med Educ. (2022) 22:322. doi: 10.1186/s12909-022-03386-8, PMID: 35473599 PMC9041277

[28] Hamdan M Hamra AA. Burnout among workers in emergency Departments in Palestinian hospitals: prevalence and associated factors. BMC Health Serv Res. (2017) 17:407. doi: 10.1186/s12913-017-2356-3, PMID: 28619081 PMC5472878

[29] Veronese G Pepe A. Psychological distress, professional burnout, and trauma in Palestinian health care helpers: a two-wave quantitative longitudinal study. Psychol Trauma. (2022) 14:523–34. doi: 10.1037/tra0000941, PMID: 34516221

[30] Mcloughlin C Abdalla A O'Callaghan AK Casey S Barrett E. The impact of COVID-19 on burnout, psychological well-being, and Work satisfaction in psychiatry trainees in Ireland. Acad Psychiatry. (2022) 46:701–9. doi: 10.1007/s40596-022-01633-0, PMID: 35441349 PMC9018053

[31] Riley MR Mohr DC Waddimba AC. The reliability and validity of three-item screening measures for burnout: evidence from group-employed health care practitioners in upstate New York. Stress Health. (2018) 34:187–93. doi: 10.1002/smi.2762, PMID: 28524379

[32] Carver CS. You want to measure coping but your protocol’s too long: consider the brief COPE. Int J Behav Med. (1997) 4:92–100. doi: 10.1207/s15327558ijbm0401_6, PMID: 16250744

[33] Kabunga A Kigongo E Okalo P Udho S Grace AA Tumwesigye R. Burnout and coping mechanisms among healthcare professionals in Central Uganda. Front Psych. (2024) 15:1373743. doi: 10.3389/fpsyt.2024.1373743, PMID: 38686129 PMC11056560

[34] Menaldi SL Raharjanti NW Wahid M Ramadianto AS Nugrahadi NR Adhiguna GMYP. Burnout and coping strategies among resident physicians at an Indonesian tertiary referral hospital during COVID-19 pandemic. PLoS One. (2023) 18:e0280313. doi: 10.1371/journal.pone.0280313, PMID: 36662883 PMC9858322

[35] Hagihara A Murata S. Burnout among physicians in Poland. Pol Arch Intern Med. (2021) 131:612–4. doi: 10.20452/pamw.16068, PMID: 34463080

[36] Hosseini SM Hesam S Hosseini SA. Burnout among military personnel: a systematic review. Iran J Psychiatry. (2023) 18:213–36. doi: 10.18502/ijps.v18i2.12371, PMID: 37383961 PMC10293693

[37] Wang Z Xie Z Dai J Zhang L Huang Y Chen B. Physician burnout and its associated factors: a cross-sectional study in Shanghai. J Occup Health. (2014) 56:73–83. doi: 10.1539/joh.13-0108-OA, PMID: 24430838

[38] Hu NC Chen JD Cheng TJ. The associations between long working hours, physical inactivity, and burnout. J Occup Environ Med. (2016) 58:514–8. doi: 10.1097/JOM.0000000000000715, PMID: 27158959

[39] Wang J Wang W Laureys S Di H. Burnout syndrome in healthcare professionals who care for patients with prolonged disorders of consciousness: a cross-sectional survey. BMC Health Serv Res. (2020) 20:841. doi: 10.1186/s12913-020-05694-5, PMID: 32894132 PMC7487695

[40] Duli S. Proactive coping and professional burnout in special education. AJIS. (2015) 4:18. doi: 10.5901/ajis.2015.v4n3s1p18

[41] Oser CB Biebel EP Pullen E Harp KL. Causes, consequences, and prevention of burnout among substance abuse treatment counselors: a rural versus urban comparison. J Psychoactive Drugs. (2013) 45:17–27. doi: 10.1080/02791072.2013.763558, PMID: 23662328 PMC3652635

[42] Bakker AB Demerouti E. Job demands–resources theory: taking stock and looking forward. J Occup Health Psychol. (2017) 22:273–85. doi: 10.1037/ocp0000056 27732008

[43] Habibisaravi R Ghasemihamedani F Oveis G Azizi S Assadi T. Assessing job burnout status in front-line healthcare providers at Mazandaran University of Medical Sciences during the COVID-19 epidemic in 2022. Health Emerg Disast Q. (2023) 8:149–58. doi: 10.32598/hdq.8.3.34.5

[44] Doolittle BR Windish DM. Correlation of burnout syndrome with specific coping strategies, behaviors, and spiritual attitudes among interns at Yale University, New Haven, USA. J Educ Eval Health Prof. (2015) 12:41. doi: 10.3352/jeehp.2015.12.41, PMID: 26201403 PMC4536357

[45] Seguin M Roberts B. Coping strategies among conflict-affected adults in low- and middle-income countries: a systematic literature review. Glob Public Health. (2017) 12:811–29. doi: 10.1080/17441692.2015.1107117, PMID: 26609735

[46] Ahmead M Shehadah F Abuiram I. Correlation of death anxiety with coping strategies among Palestinian women with breast cancer: a cross-sectional study. Front Public Health. (2024) 12:1420306. doi: 10.3389/fpubh.2024.1420306, PMID: 38915747 PMC11194404

[47] Hofmann SG Hay AC. Rethinking avoidance: toward a balanced approach to avoidance in treating anxiety disorders. J Anxiety Disord. (2018) 55:14–21. doi: 10.1016/j.janxdis.2018.03.004, PMID: 29550689 PMC5879019

[48] Cecil J McHale C Hart J Laidlaw A. Behaviour and burnout in medical students. Med Educ Online. (2014) 19:25209. doi: 10.3402/meo.v19.25209, PMID: 25160716 PMC4145104

[49] Andrade GF da Silva Menolli PV Clemente PA Mesas AE Silva DC Girotto E. Burnout syndrome and consumption of alcohol and illicit substances in university students. Paidéia. (2021) 31. doi: 10.1590/1982-4327e3134

[50] Elkardi S Choujaa H Agoub M. Burn out of health care professionnels leads to addiction. Eur Psychiatry. (2023) 66:S466–7. doi: 10.1192/j.eurpsy.2023.1000

[51] Carlotto RC Teixeira MAP Dias ACG. Adaptação acadêmica e coping em estudantes universitários [Academic adaptation and coping in university students]. Psico USF. (2015) 20:421–32. doi: 10.1590/1413-82712015200305

[52] Pietrowski DL Cardoso NO Bernardi CCN. Estratégias de coping frente à síndrome de burnout entre os professores: uma revisão integrativa da literatura nacional. Cont Clín. (2018) 11:397–409. doi: 10.4013/ctc.2018.113.10

[53] Winkel AF Woodland MB Nguyen AT Morgan HK. Associations between residents’ personal behaviors and wellness: a national survey of obstetrics and gynecology residents. J Surg Educ. (2020) 77:40–4. doi: 10.1016/j.jsurg.2019.08.014, PMID: 31492641

[54] Allen HK Lilly F Green KM Zanjani F Vincent KB Arria AM. Graduate student burnout: substance use, mental health, and the moderating role of advisor satisfaction. Int J Ment Health Addict. (2022) 20:1130–46. doi: 10.1007/s11469-020-00431-9 35400127 PMC8992873

[55] Kusalaruk P Saravithi J. Coping strategies and relationship with burnout among residents in Thailand. Eur Psychiatry. (2023) 66:S321–2. doi: 10.1192/j.eurpsy.2023.711

[56] Gibbons C. Stress, coping, burnout and mental health in the Irish police force. J Police Crim Psych. (2024) 39:348–57. doi: 10.1007/s11896-023-09638-7

[57] Navinés R Olivé V Fonseca F Martín-Santos R. Work stress and resident burnout, before and during the COVID-19 pandemia: an up-date. Med Clin. (2021) 157:130–40. doi: 10.1016/j.medcli.2021.04.003, PMID: 35005240 PMC8721440

